# Assessing and Minimizing Re-identification Risk in Research Data Derived from Health Care Records

**DOI:** 10.5334/egems.270

**Published:** 2019-03-29

**Authors:** Gregory E. Simon, Susan M. Shortreed, R. Yates Coley, Robert B. Penfold, Rebecca C. Rossom, Beth E. Waitzfelder, Katherine Sanchez, Frances L. Lynch

**Affiliations:** 1 Kaiser Permanente Washington Health Research Institute, Seattle, WA, US; 2 HealthPartners Institute, Minneapolis, MN, US; 3 Kaiser Permanente Hawaii Center for Health Research, Honolulu, HI, US; 4 Baylor Scott and White Research Institute, Dallas, TX, US; 5 Kaiser Permanente Northwest Center for Health Research, Portland, OR, US

**Keywords:** privacy, electronic health records, confidentiality, HIPAA, data sharing

## Abstract

**Background:**

Sharing of research data derived from health system records supports the rigor and reproducibility of primary research and can accelerate research progress through secondary use. But public sharing of such data can create risk of re-identifying individuals, exposing sensitive health information.

**Method:**

We describe a framework for assessing re-identification risk that includes: identifying data elements in a research dataset that overlap with external data sources, identifying small classes of records defined by unique combinations of those data elements, and considering the pattern of population overlap between the research dataset and an external source. We also describe alternative strategies for mitigating risk when the external data source can or cannot be directly examined.

**Results:**

We illustrate this framework using the example of a large database used to develop and validate models predicting suicidal behavior after an outpatient visit. We identify elements in the research dataset that might create risk and propose a specific risk mitigation strategy: deleting indicators for health system (a proxy for state of residence) and visit year.

**Discussion:**

Researchers holding health system data must balance the public health value of data sharing against the duty to protect the privacy of health system members. Specific steps can provide a useful estimate of re-identification risk and point to effective risk mitigation strategies.

## Introduction

A growing emphasis on data sharing in the scientific community is colliding with increasing concerns among the general public and health care systems regarding data privacy. Sharing of data at time of research publication supports the rigor and reproducibility of research. Availability of data for secondary analyses can accelerate discovery and development of new methods. The National Institutes of Health and other funders of research increasingly expect sharing of final research data [1] and provide resources or tools to facilitate wide access to research data [2]. But recent highly publicized privacy breaches and instances of data misuse have increased attention to risks of sharing sensitive and potentially identifiable information [3,4,5]. Investigators who would hope to share research data must attempt to balance these competing values.

An extensive literature [3,6,7,8,9] describes underlying theory and specific mathematical methods for assessing risk that individuals in a potentially sensitive dataset could be identified by linkage to publicly available data. In this paper, we attempt to summarize that theory and those methods for non-technical users, such as health system Privacy Offices and Institutional Review Boards. We discuss specific aspects of health research datasets that create risk of re-identification through linkage with other data sources. We describe a concrete framework for assessing and mitigating risk of re-identification prior to sharing sensitive research data derived from health records. We illustrate use of that framework by applying it to a large dataset including detailed health records linked to data regarding suicidal behavior – proposing a specific risk mitigation strategy for that example. We focus primarily on risks related to linkage of research data to large public data resources, but we also consider risk of efforts focused on re-identifying specific individuals.

## Example: Suicide Risk Prediction Data

The tension between public health benefit and data privacy is especially sharp in research regarding suicide risk. The Health Care Systems Research Network’s Mental Health Research Network (MHRN) has recently described development and validation of models predicting risk of suicidal behavior following an outpatient visit to a mental health specialty provider or a primary care visit with a mental health diagnosis. Data from seven MHRN health systems identified approximately 20 million eligible visits (by approximately 3 million health system members) between 2009 and 2015. Data used to develop and validate prediction models include records of suicidal behavior and discrete indicators of prior mental health and general medical diagnoses and treatments, all extracted from the HCSRN Virtual Data Warehouse tables in each participating health system [10].

Potential predictors of suicidal behavior considered in this work included demographic characteristics (five age categories, sex, race, ethnicity), current and past mental health and substance use diagnoses (organized in 12 categories), past suicide attempts, other past injury or poisoning diagnoses, dispensed prescriptions for mental health medication (organized in four categories), past inpatient or emergency department mental health care, general medical diagnoses (by Charlson Comorbidity Index [11] categories), and recorded scores on the Patient Health Questionnaire (PHQ-9) depression scale [12] used routinely in these health systems. Clinical predictors were represented as dichotomous indicators. Each diagnosis category was represented by three overlapping indicators (recorded at or within 90 days prior to the visit, recorded within one year prior, and recorded within five years prior). Each category of medication or emergency/inpatient utilization was represented by three overlapping indicators (occurred within 90 days prior to the visit, one year prior, or any time prior). To represent temporal patterns of prior PHQ-9, 24 indicators were calculated for each encounter to represent number of observations, maximum value, and modal value (including value of missing) during three overlapping time periods (previous 90 days, previous 183 days, and previous 365 days). The final set of potential predictors for each encounter included 149 indicators. These data were used to develop and validate model to calculate risk scores for suicide attempt and suicide death in the 90 days following an outpatient visit.

Performance of these prediction models substantially exceeds previous efforts to predict suicidal behavior [13,14,15]. Risk prediction scores are also substantially more accurate than currently available brief screening questionnaires [16,17].

Notwithstanding the accuracy and utility of these risk prediction tools, further improvements in prediction accuracy are certainly possible. Alternative model-fitting methods could circumvent the need for analysts to specify categories or time periods in advance. Alternative analytic methods could also identify potentially informative effect modification among tens of thousands of potential interaction terms. Sharing these data with a wide range of analysts and methods developers (including those working in other scientific areas), could significantly accelerate progress in development of new methods to address important analytic challenges such as correlation between observations for a single individual and selection biases due to patient driven timing of visits.

In addition, sharing of this data resource would also allow secondary analyses addressing important substantive questions. Additional model development and validation work could include models limited to specific patient groups or models limited to data elements available in specific health care organizations. The large sample size and wide range of predictors could also support analyses regarding more traditional inferential questions. For example, secondary analysis could examine racial and ethnic differences in risk of suicide attempt to explore cultural differences in impact of specific clinical risk factors [18].

Ideally, a public use data set would be shared with any interested researchers or developers of analytic methods. Sharing data under more restrictive mechanisms, such as by formal data use agreements or through a restricted data enclave, would restrict the pool of researchers or analysts able to access data and would require significant resources for ongoing administration. Given the sensitivity of data regarding mental health history and suicidal behavior, any plans to share a public use dataset must carefully consider risk that individuals contributing data could be re-identified. Because re-identification of any individual would be a serious breach of trust, we are concerned with the maximum (rather than average) risk of re-identification for individuals in the dataset. We describe below a systematic approach for assessing and mitigating risk of re-identification when sharing such sensitive health information.

## Legal and Regulatory Requirements

The Health Insurance Portability and Accountability Act (HIPAA) established regulatory thresholds for sharing of data from health records. By HIPAA standards, a de-identified dataset contains no direct or known indirect identifiers. Risk of re-identification is presumed to be very low. Data judged to be de-identified can be shared without a formal Data Use Agreement or any other legal or technical restrictions on access. By HIPAA standards, a limited dataset contains potential indirect identifiers. Risk of re-identification is presumed to be greater than “very small” (the HIPAA standard for de-identification). HIPAA requires that sharing of a limited dataset (including potential identifiers) must be governed by a formal Data Use Agreement or by technical restrictions to prevent re-identification (such as a data enclave with restrictions on allowed queries).

HIPAA specified two mechanisms for assessing identifiability of electronic health data: the Safe Harbor standard and the Expert Determination standard [19]. Under the Safe Harbor standard, a dataset derived from health records can be considered de-identified if it contains none of 18 specified direct or indirect identifiers (e.g., zip code, date of service, telephone number) [19]. To satisfy the Safe Harbor standard, the data steward must also have no “actual knowledge” that other data elements not specified in the law could serve as indirect identifiers. Under the Expert Determination standard, a person with knowledge of the data and appropriate statistical expertise must determine that the risk of re-identification is “very small.” Theoretically, a determination under the Expert Determination standard could be more or less strict than application of the Safe Harbor standard. An expert could determine that data elements forbidden under the Safe Harbor standard (e.g., dates more precise than calendar year) could be released while maintaining a “very small” risk of re-identification. Alternatively, an expert with knowledge of the data and knowledge of available external data sources could determine that additional restrictions (over and above those required by the Safe Harbor standard) are necessary.

The MHRN suicide risk prediction dataset was designed to satisfy the HIPAA Safe Harbor standard for de-identification. Outcome and predictor variables do not include any of the 18 specified potential identifiers. Date of index visit is indicated by calendar year only. Dates of all other predictor and outcome variables are expressed in relation to (days before or days after) the index visit date. Predictors include some census-derived indicators of neighborhood socioeconomic status (median income, median educational attainment), but original geographic indicators are not included. Multiple visits per person are linked by a randomly generated person identifier rather than by health plan number. The project team has no actual knowledge of other potential direct or indirect identifiers.

While we can have no “actual knowledge” regarding clear routes to re-identification, our responsibility to health system members does obligate us to consider less obvious sources of re-identification risk. We are certainly aware of previous attempted and successful re-identification attacks on public health care databases [3]. And we are certainly aware of methods for assessing risk of re-identification using combinations of variables, any one of which individually would not create significant risk. A successful re-identification attack on a public dataset regarding suicide risk (or some other sensitive health condition) could harm patients and seriously damage the reputations of our partner health care systems, prompting more restrictive policies regarding waiver of consent for research use of health records data.

## Sources of Re-identification Risk

Assessments of re-identification risk have sometimes focused on the distribution or “cell sizes” of individual variables or data elements. A cell size or count of 5 or 6 is often held out as a threshold for unacceptable risk of re-identification. Policies of some research institutions (including some HCSRN research centers) often call for suppression of variables with counts below this threshold or for suppression of such small cell sizes in tabulations or other summary reports.

The distribution of any single variable, however, is not an accurate indicator of re-identification risk. In relatively small datasets, even common indicators such as race or age group may have frequencies below 5 or 6. But, for example, a disclosure that only 4 of 200 individuals in a sensitive research dataset are classified as Native American does not in and of itself create risk of re-identification. Conversely, reliance on distribution of single variables to assess re-identification risk in large datasets may be falsely reassuring. In our suicide risk prediction dataset, even the sparsest and most sensitive variables, such as diagnosis of suicide attempt in the 90 days before an index visit, have counts greater than 1,000 in every participating health system. But that large cell size certainly does not imply that data regarding recent suicide attempts could not, in combination with other data elements, create risk of re-identification.

The uniqueness of any record depends not on the distribution or frequency of any single variable but rather on the potential cross-classification of multiple variables. In a dataset containing even a moderate number of variables, a large proportion of records will be completely or relatively unique. For example, 20 dichotomous variables define an array of over one million permutations or cells. Even in a sample of millions of records, many of those cells will have counts smaller than 5 or 6, including large numbers of cells identifying a single record.

Risk of re-identification, however, depends on uniqueness in relation to identifiable external data sources. Data sources available to the general public, for free or for purchase, include voter registration lists, birth or death records, consumer purchase data, and credit data. External data sources including partial health information could allow linkage to more detailed and sensitive health information. For example, an identified database of general hospital discharges could be linked to more detailed data regarding mental health treatment. To assess re-identification risk in relation to any specific external data source, we must consider potential uniqueness of records with cells or classes defined by data elements included in both the sensitive research dataset and a public or private data resource available to an “adversary” who might attempt to link the two. It is the combination of uniqueness and availability that creates re-identification risk. To use a financial analogy, the exact amount (in dollars and cents) of the last 5 transactions in any credit account may be unique, but it would only be identifying to an adversary who already had access to those banking records.

Paradoxically, re-identification risk is most often created by the least sensitive data elements in a sensitive dataset. While highly sensitive data elements (such as the mental health diagnosis and treatment indicators in our suicide risk prediction dataset) could define small groups of individuals, those data elements would not be available to anyone who did not already possess comprehensive health records. More widely available and less sensitive data (demographic characteristics, births, deaths) are more likely to create new opportunities to re-identify sensitive information.

**Figure 1:**
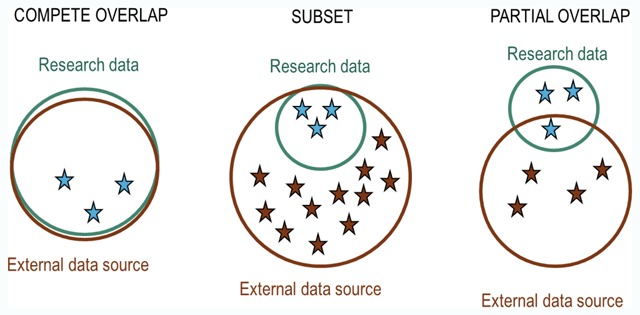
Possible relationships between populations covered by sensitive research dataset and identified external dataset. Stars represent a small cell or class of individuals defined by data elements common to two datasets.

Risk of re-identification also depends on the pattern of overlap between the population covered by a research dataset and the population covered by an external data source to which it might be linked. Three scenarios are illustrated in Figure 1. Risk is highest, but easiest to estimate, if the population covered by research dataset and the population covered by an external data source are congruent or completely overlapping. An example of this scenario would be linking a state-level all-payer insurance claims database to an identified state database of motor vehicle crashes. If some combination of overlapping variables (age, sex, zip code, accident or injury date) identifies three individuals in the detailed insurance claims data, then linkage would identify exactly those three individuals in the motor vehicle data. And each of those individual’s health records could be identified with a certainty of 1 in 3.

Risk is lower, but also more difficult to estimate, if the private or sensitive database represents only a subset of the population of the public or less sensitive database. An example of this scenario would be linking the same statewide motor vehicle crash database to claims records from a single insurer covering approximately 1 in 5 state residents. If overlapping variables identified a class of three individuals in the research database, then linkage would identify an estimated 15 corresponding records in the statewide data. Each of the three individuals would be linked, and each of those individual’s health records could be identified with a certainty of 1 in 15.

Risk is lower, and even more difficult to estimate if the two data sources cover partially overlapping populations. An example of this scenario would be linking the same statewide motor vehicle database to detailed claims records from an insurer coving approximately 1 in 5 residents in a 3-state area. If overlapping variables identified a class of three individuals in the sensitive research database, then linkage would connect an estimated 1 of those individuals to an estimated 5 in the state motor vehicle database. On average, only one of three individuals would be linked, and records for that individual could be identified with a certainty of 1 in 15. While each scenario involves a class or cell size of 3 in the sensitive research database, quantitative risk of re-identification varies according to the pattern of overlap.

The logic presented above illustrates how re-identification risk for any individual record depends on the availability of external data with overlapping data elements, the size of the class defined by those overlapping elements in which the record in question falls, and the overlap in the population covered by the research dataset and the population covered by the external data source. More technical discussions of this topic [6] provide specific methods for calculating overall and record-level risk.

The discussion above focuses on estimating group-level risk created by linkage to large public or semi-public data resources, rather than on re-identification attempts focused on individuals. Potential examples of individual-level re-identification attempts would include identifying a high-profile individual in a research dataset based on information in news reports regarding a specific health care incident [20] or identification of a co-worker in a research dataset based on personal knowledge of a specific treatment event. The general principles described above do apply, but estimation of risk may be more challenging. Some of the potential linking elements in those individual cases would correspond to risk-creating elements in large public databases (age, sex, race, ethnicity).

News reports or other sources of personal knowledge carry an additional potential risk. Those individual-level descriptions might also contain idiosyncratic data elements more difficult to anticipate or estimate [20]. To illustrate with an hypothetical example related to suicide risk: a press account of a suicide attempt by a high-profile individual might include information regarding specific and uncommon mental health or medical diagnoses, specific treatments received, or means of self-harm. Those additional data elements could uniquely identify an individual in a large research dataset. While it is not possible to anticipate every type of information available through press accounts or personal knowledge, we can say that re-identification risk is higher when a research dataset includes information regarding rare diagnoses or more highly visible health care events (such as hospitalizations or emergency department encounters).

## Framework for Assessment of Re-identification Risk

The logic described above suggests a three-step framework for assessing risk of re-identification through linkage to identified external data.

First, a data steward should consider the range of external data sources containing overlapping data elements as well as explicit identifiers. Those overlapping data elements might include demographic characteristics (e.g., age, sex, race, ethnicity), location characteristics (e.g., state, county) and/or a subset of health-related data elements (e.g., a specific diagnosis or type of service use in a specific year). Those common data elements linked to explicit identifiers (name, address, Social Security number) create risk of re-identification. In the case of the MHRN suicide risk prediction dataset, the most obvious such external data sources would be state mortality data derived from death certificates. Overlapping data elements in suicide risk prediction datasets and state mortality records would include age group, sex, race, ethnicity, year of death, and injury or poisoning diagnoses recorded as causes of death. Commercially available databases of consumer purchases might include age, sex, race, ethnicity, and dates of payments to health care providers or organizations.

**Figure 2:**
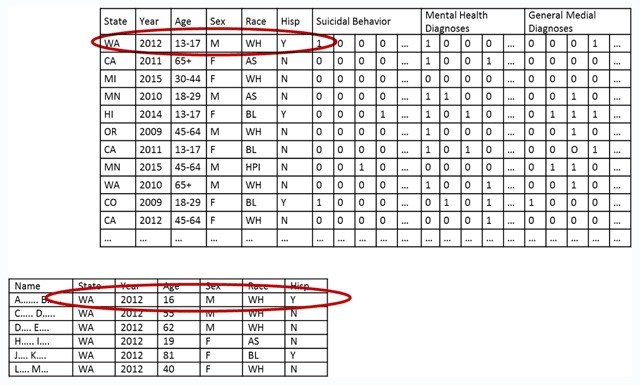
Potential linkage of suicide risk prediction dataset to state mortality records using shared data elements.

Second, a data steward should examine the prevalence of unique or nearly unique records defined by those overlapping data elements. These are the “small cell sizes” that allow definite or probable linkage to explicit identifiers in an external data source. In the case of the MHRN suicide risk prediction dataset, the data elements overlapping with any state mortality database would include sex, five age groups, six race categories, Hispanic ethnicity, calendar year of death, and broad category of cause of death diagnosis (e.g., poisoning classified as having undetermined intent). Figure 2 illustrates identification of an individual record using a unique combination of these overlapping variables. Straightforward programming or available software [21] can identify the number of cells or classes below any specific count threshold (sometimes referred to as a k-anonymity threshold) [22].

Third, a data steward should consider the likely pattern of population overlap with identified external data sources. If the population covered by a research database overlaps completely with that covered by an external data source, then each cell or class in the research database can be completely linked to the corresponding cell or class in the external database. Each individual in a cell or class with *n* members could be identified with a certainty of *1/n*. If the population covered by the research database is a random subset (probability *p_1_*) of the population covered by external data, then then records for *n* individuals in the research dataset would be linked to approximately *n/p_1_* records in identified external data. Each individual in that class in the research database could be identified with an estimated certainty of *p_1_/n*. If only a random subset (probability *p_2_*) of the population covered by the research database is also covered by the external database and if *p_1_* of the population covered by the external database is covered by the research database (i.e., partial overlap scenario), then each individual in a class of size *n* in the research database could be identified with an estimated certainty of *p_1_p_2_/n*.

The calculations above depend on some simplifying assumptions. First, we assume that the distributions of overlapping or linking data elements (e.g., race, sex) are similar in the sensitive research dataset and the identifiable external data source. If not, then re-identification probabilities could be considerably higher. More sophisticated calculations consider those distributions in both data sources when estimating sizes of cells or classes defined by overlapping data elements [6]. Second, we assume that the characteristics defining overlap (e.g., insurance plan membership) are not present in the external data source. If that overlap-defining characteristic were available, then the subset and partial overlap scenarios would devolve to the higher-risk complete overlap scenario.

**Figure 3:**
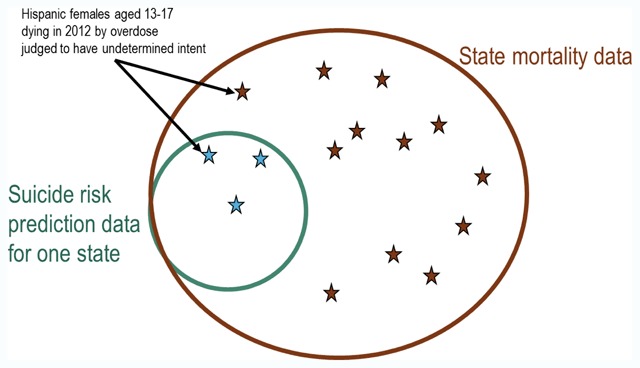
Relationship of specific small cell in suicide risk prediction dataset to matching records in state mortality data.

Figure 3 illustrates the application of these 3 steps to our suicide risk prediction dataset to estimate re-identification risk due to linkage with identified state mortality data. We consider the hypothetical scenario in which a specific combination of data elements common to the two datasets (Hispanic females aged 13–17 dying in 2012 in Washington state by overdose judged to have undetermined intent) identifies only 3 individuals in the suicide risk prediction dataset. If we assume that Kaiser Permanente of Washington covers 20 percent of the state population, then those three records in the research dataset would match to an estimated total of 15 records in Washington state mortality data. As described above, these estimates assume that overlapping data elements (e.g., race or ethnicity) have similar distributions in the research dataset as in state mortality records.

## Framework for Mitigating Re-identification Risk

If it is possible to directly examine relevant external data, then risk can be addressed directly at the individual record level. A data steward could identify each record in the research dataset that can be linked (either definitively or with a probability exceeding a specific threshold) to a record in the external data source. Strategies for mitigating risk at the individual record level include deleting “high-risk” records or altering values of overlapping variables in high-risk records to eliminate unique or small-cell matches. Any of these alterations could, of course, reduce the scientific or public health value of a public-use dataset. But a minimally altered public-use dataset could still have significant public health value. A data steward considering alternative alteration strategies should attempt to anticipate future use and avoid or minimize alterations that would compromise answers to the highest-value questions. Rationale for and specific locations of alterations should also be transparent to all users.

If it is not possible to directly examine relevant external data, then risk mitigation must more often involves modifying overlapping variables or columns rather than modifying individual records or rows. Strategies for mitigating risk at the variable level would include deletion of overlapping variables (i.e., variables found in the sensitive research dataset and an identified external data source) as well as collapsing, blurring, or distorting categories of overlapping variables. In large datasets, regression approaches can facilitate identifying specific sources of risk. For example, after aggregating the data to obtain a count of unique rows (i.e., records that share the same values for potentially overlapping data elements). Poisson regression could be used to identify variables associated with low counts or small classes.

In the case of linking our suicide risk prediction dataset to state mortality data, we are not able to directly examine state mortality records to directly identify high-risk records. Participating health systems are able to link membership records to state mortality data in order to ascertain suicide attempts. But researchers are not permitted to search for matching records in the complete state populations.

**Figure 4:**
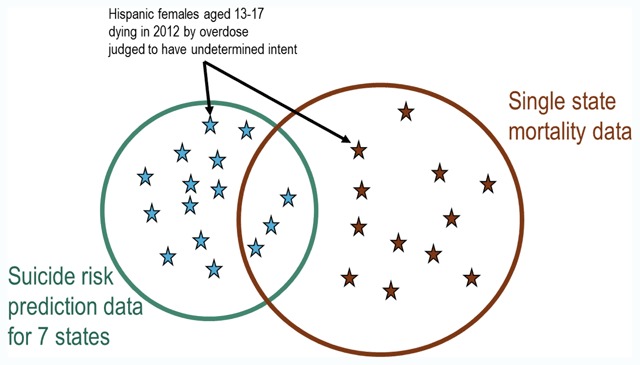
Relationship of specific small cell in suicide risk prediction dataset to matching records in state mortality data after exclusion of health system (i.e., state) identifier.

**Figure 5:**
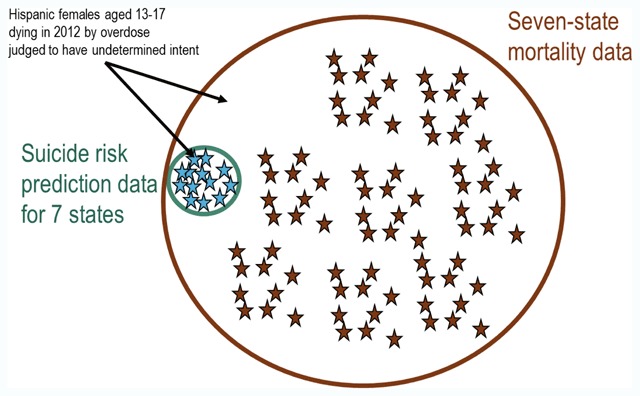
Relationship of specific small cell in suicide risk prediction dataset to matching records in national mortality data after exclusion of health system (i.e., state) identifier.

Consequently, we must consider broader risk mitigation strategies rather than strategies limited to directly identified specific records. Overlapping variables include age, sex, race, ethnicity, health system (i.e., state of residence), and cause of death diagnosis (in broad categories). Deleting, collapsing, or otherwise altering data regarding cause of death would certainly reduce scientific or public health value of a resulting public-use dataset. Similarly, age, sex, race, and ethnicity are all important predictors of suicidal behavior. Of overlapping variables, health system (or state of residence) probably carries the lowest public health value. While rates of suicidal behavior do vary across health systems (mirroring well-established differences across U.S. regions), analyses of differences among these seven health systems would contribute little to generalizable knowledge. Deletion of the health system/state variable would reduce re-identification risk in two ways. First it would reduce the number of small cells or classes in the research dataset, since small cells previously distinguished by the health system variable would be combined. Second, it would change the pattern of overlap between the suicide risk prediction dataset and external mortality records. If state of residence were no longer included in the suicide risk dataset, then the overlap with any state mortality database would shift from a subset scenario (Figure 3) to a lower-risk partial overlap scenario (Figure 4). While the suicide risk dataset would remain a subset of data from all seven states, suicide deaths in the risk prediction dataset would account for approximately 15 percent of a much larger total seven-state (Figure 5).

While we assume that removing the health system or state variable would significantly increase the sizes of the smallest cells defined by potentially linkable data elements (i.e., increase k-anonymity threshold), this assumption should be formally tested. If some of those potentially linkable elements (e.g., race or ethnicity) are unevenly distributed across health systems or states, then the reduction in risk may be smaller than predicted.

## Discussion

We describe above a framework for assessing and mitigating risk of re-identification when sharing research data derived from health care records. We emphasize that risk of re-identification depends on the overlap of data elements in a sensitive research database and some identified or identifiable external data resource. Specifically, risk depends on the size of cells or classes defined by those overlapping data elements as well as the pattern of overlap between the population covered by the research database and that covered by the external data source. If an external data source is directly available, then a data steward can directly identify high-risk records and mitigate risk at the individual record level. If an external data source is not available, then risk mitigation strategies will more often focus on deletion or modification of specific variables or data elements.

There is, of course, no universal threshold for an acceptable risk of re-identification. While we can attempt to estimate expected risk, the likelihood of a successful re-identification attack also depends on the motivation of and resources available to a potential adversary. Those external factors may not be known at the time of data release. Some previous work on re-identification risk has used the expected cost of a re-identification attack to calibrate acceptable risk level (including use of game theory to explicitly balance cost of protection efforts against cost of a re-identification attack). But those who would hope to re-identify individuals may have non-financial motivations. Law and regulatory guidance intentionally do not provide a quantitative threshold for “very small” risk of re-identification. The acceptability of small risks will likely vary among individual patients and different health care systems. Data stewards must eventually make well-considered decisions balancing estimates of re-identification risk, the sensitivity of health information that might be disclosed, and the public health value of releasing data for legitimate scientific uses.

Our framework does not address some other risks to health care systems from sharing of research data. Release of detailed data regarding mental health history and suicidal behavior could expose health systems to inappropriate or ill-informed attempts to compare suicide attempt rates or patterns of mental health care across health systems. In some other cases, release of detailed data on treatment patterns could reveal proprietary information regarding staffing levels or purchasing decisions. Data stewards must also consider these risks, and different remedies to reduce these risks may be necessary.

Our example considers risk related to a single external data source, state or national mortality records. Other health-related data sources (such as a state-level insurance claims database or a hospital discharge database) would create different patterns of risk and might require different mitigation strategies. Other types of population-based data (such as consumer purchase data) would also raise different specific challenges. We believe, however, that the general framework we describe would be applicable to a wide range of external data sources.

As is clear from the illustrations above, risk of re-identification does not fall equally among all people included in a research dataset. Instead it falls largely or exclusively on those in small cells or classes, usually defined by demographic or health-related characteristics. Consequently, risk may fall disproportionately on vulnerable or traditionally disadvantaged groups, including people with rarer health conditions and members of minority racial or ethnic groups. When evaluating risks of data sharing to those vulnerable or disadvantaged groups, data stewards should consider this disproportionate burden as well as the benefits to those groups from inclusion in research using shared data.

After making best efforts to assess and reduce re-identification risk, proponents of data sharing still face a challenging communication task. Maintaining the trust of health systems and patients will require effective communication regarding risks to privacy and benefits of research. Potential benefits of data sharing are broadly distributed, but risks or potential harms are localized to specific health systems and their members or patients. If we were explaining the situation illustrated by Figure 3 to a family member of a suicide decedent, we could say “Someone with both the research data and the state death certificate data could find 15 sets of health records in the research data and know that your daughter was one of those 15. Sharing this information with other researchers won’t help your daughter, but it might help someone like her.”
